# Single START-domain protein Mtsp17 is involved in transcriptional regulation in *Mycobacterium smegmatis*

**DOI:** 10.1371/journal.pone.0249379

**Published:** 2021-04-15

**Authors:** Ying Zhou, Tianying Zhong, Wenjing Wei, Zhuhua Wu, Anping Yang, Ning Liu, Ming Wang, Xiaoli Zhang

**Affiliations:** 1 Department of Bone and Joint Surgery, The First Affiliated Hospital of Jinan University, Guangzhou, China; 2 Guangdong Province Green and High Performance Novel Materials Engineering Research Center, Jiangmen Polytechnic, Jiangmen, China; 3 Center for Tuberculosis Control of Guangdong Province, Guangzhou, China; 4 School of Medicine, Foshan University, Foshan, Guangdong, China; 5 Key Laboratory of RNA Biology, Institute of Biophysics, Chinese Academy of Sciences, Beijing, China; Bose Institute, INDIA

## Abstract

Tuberculosis caused by the pathogen *Mycobacterium tuberculosis* (MTB), remains a significant threat to global health. Elucidating the mechanisms of essential MTB genes provides an important theoretical basis for drug exploitation. Gene *mtsp17* is essential and is conserved in the Mycobacterium genus. Although Mtsp17 has a structure closely resembling typical steroidogenic acute regulatory protein-related lipid transfer (START) family proteins, its biological function is different. This study characterizes the transcriptomes of *Mycobacterium smegmatis* to explore the consequences of *mtsp17* downregulation on gene expression. Suppression of the *mtsp17* gene resulted in significant down-regulation of 3% and upregulation of 1% of all protein-coding genes. Expression of *desA1*, an essential gene involved in mycolic acid synthesis, and the anti-SigF antagonist MSMEG_0586 were down-regulated in the conditional Mtsp17 knockout mutant and up-regulated in the Mtsp17 over-expression strain. Trends in the changes of 70 of the 79 differentially expressed genes (Log_2_ fold change > 1.5) in the conditional Mtsp17 knockout strain were the same as in the SigF knockout strain. Our data suggest that Mtsp17 is likely an activator of *desA1* and Mtsp17 regulates the SigF regulon by SigF regulatory pathways through the anti-SigF antagonist MSMEG_0586. Our findings indicate the role of Mtsp17 may be in transcriptional regulation, provide new insights into the molecular mechanisms of START family proteins, and uncover a new node in the regulatory network of mycobacteria.

## Introduction

Mtsp17 (Rv0164) is a 17-kDa protein isolated from *Mycobacterium tuberculosis* (MTB) culture filtrates that has been shown to be a T-cell and B-cell antigen [1,2]. It is highly conserved across the Mycobacteria. MTB Mtsp17 shares less than 30% homology with its homologues in other genera, making it not only an interesting anti-TB drug target but also an immunogen that may be worth investigating in vaccine development. Although *mtsp17* has been shown to be an essential gene in both *Mycobacterium smegmatis* (MSM) and MTB [3,4], its physiological function is presently unclear. Considering its essentiality in the genus Mycobacterium, further investigation of Mtsp17 will likely provide new insights into the physiology of Mycobacteria.

Mtsp17 folds in a similar manner to polyketide cyclases/aromatases (CYC/ARO), even though they share less than 20% sequence identity [3]. CYC/ARO proteins are monofunctional enzymes in Type II polyketide synthase (PKS) complexes and transform linear poly-*β*-ketone intermediates into aromatic polyketides [5]. Twenty-one Type I and three Type III PKSs have been annotated in Mycobacteria, but Type II PKS proteins have not yet been identified [6]. Structural comparisons and docking studies have suggested that Mtsp17 is likely not an enzyme that catalyzes polyketide cyclization and aromatization [3]. The essential functions of Mtsp17 in mycobacteria are therefore likely to operate by mechanisms that are different from CYC/ARO proteins.

Mtsp17 is a single-domain steroidogenic acute regulatory protein-related lipid transfer (START) domain protein. START domains are lipid-binding domains that function as polyketide cyclases/aromatases in lipid metabolism. START domains also bind ligands during non-vesicular traffic between intracellular compartments and fuse with DNA binding domains to modulate gene transcription [7–9]. As mycobacteria lack internal organelles such as the endoplasmic reticulum and Golgi apparatus, the START domain in Mtsp17 may regulate transcription through a DNA contact-independent mechanism. Comprehensive profiling of the genes and pathways regulated by Mtsp17 should provide insights into its physiological functions.

In this study, we performed RNA-sequencing (RNA-seq) based transcriptomics analysis to obtain a global profile of genes regulated by Mtsp17 in MSM. We compared the transcriptomes of wildtype and the *mtsp17*-complemented strains (*mtsp17* induced and not induced), and discovered that Mtsp17 has transcriptional regulatory properties. This transcriptional regulatory role was further validated by qPCR and its implications are discussed.

## Materials and methods

### Bacterial strains and culture conditions

As knockout of *mtsp17* was lethal in MSM, a conditional Mtsp17 knockout strain (M0129C) was created by a specialized transduction procedure described previously [3] in which plasmid-encoded Mtsp17 was induced by tetracycline while the genomic *mtsp17* gene was deleted (M0129C was also the *mtsp17*-complemented strain). Over-expression of Mtsp17 was accomplished with a pMV261 plasmid that constitutively over-expressed Mtsp17 using a Hsp60 promoter [10]. *Mycobacterium smegmatis* strains were cultured in Middlebrook 7H9 medium (Difco, Baltimore, MD, USA) supplemented with 0.5% glycerol and 0.05% Tween 80 at 37°C. 25 μg^.^mL^-1^ kanamycin and 20 ng^.^mL^-1^ tetracycline (Sangon Biotech, Shanghai, China) were required for culturing the *mtsp17*-complemented strain in which the genomic *mtsp17* gene was replaced by a sacB-hyg cassette and an extrachromosomal *mtsp17* gene was introduced using a pMind-derived plasmid. After cultures of the complemented strain reached exponential phase in the presence of tetracycline (OD_600_ = 0.4), they were transferred into fresh culture medium and divided into two halves. Tetracycline (20 ng^.^mL^-1^) was added to one half of the culture (M0129C_T20, induced) and incubated for 4 hours, while the other half was incubated without tetracycline (M0129C_T0, not induced) for 4 more hours. Total RNA was then isolated from these two cultures of the complemented strain for sequencing. An exponential phase culture of *Mycobacterium smegmatis* mc^2^155 was used as a control (Wt).

### RNA isolation and transcriptome analysis

10 ml of exponentially growing cultures was harvested by centrifugation (4°C, 3000 rpm, 10 min). Total RNA was isolated from three biological replicates of each sample using a FastPrep Instrument and FastRNA Pro Blue Kits (MP Biomedicals, Solon, OH, USA) according to the manufacturer’s instructions. After treatment with DNase I, the RNA was precipitated with ammonium acetate/isopropanol. The quality and quantity of each RNA sample were assessed using an Agilent 2100 Bioanalyzer (Agilent Technologies, Beijing, China) and a NanoDrop 1000 spectrophotometer (Thermofisher, Shanghai, China). A Ribo-Zero rRNA Removal Kit (Epicentre, Madison, WI, USA) was used to remove bacterial rRNA from total RNA preparations.

cDNA libraries were constructed using a NEBNextUltraTM RNA library Prep Kit (New England Biolabs, Beijing, China) and sequenced on a HiSeq 4000 sequencer (Illumina). The processed reads were mapped to the *Mycobacterium smegmatis* MC^2^ 155 genome (NC_008596) using Bowtie2 v2.2.6 with default parameters. FPKM values for each gene were compared and differentially expressed genes (DEGs) were determined with HTSeq v0.6.0 and DESeq v1.22.1. Genes with a multiple hypothesis-adjusted p-value below 0.05 (adjusted p-value < 0.05) were considered as DEGs. Functional enrichment analysis was carried out using GOseq v1.22 based on the Gene Ontology database.

### Real-time fluorescence quantification PCR (qPCR) analysis

qPCR was used to measure the expression of selected genes in a CFX96 instrument (Bio-Rad). The GoScript Reverse Transcription System (Promega, Beijing, China) was used for cDNA preparation, and a 2x SYBR Green mix (Vazyme, Nanjing, China) and gene-specific primers (S1 Table) were used to amplify the genes (PCR cycle: 95°C, 10 s; 60°C, 30 s; 40 cycles). Primers were optimized to give 90–110% amplification efficiency and had a single melting temperature. *sigA* (MSMEG_2758) was used as an internal normalization standard. Relative quantification was performed using the ΔΔCT method. Fold changes presented are means ± standard deviations of three technical replicates.

## Results

### RNA-sequencing results uncover the importance of Mtsp17 in transcription regulation

**Fig 1:**
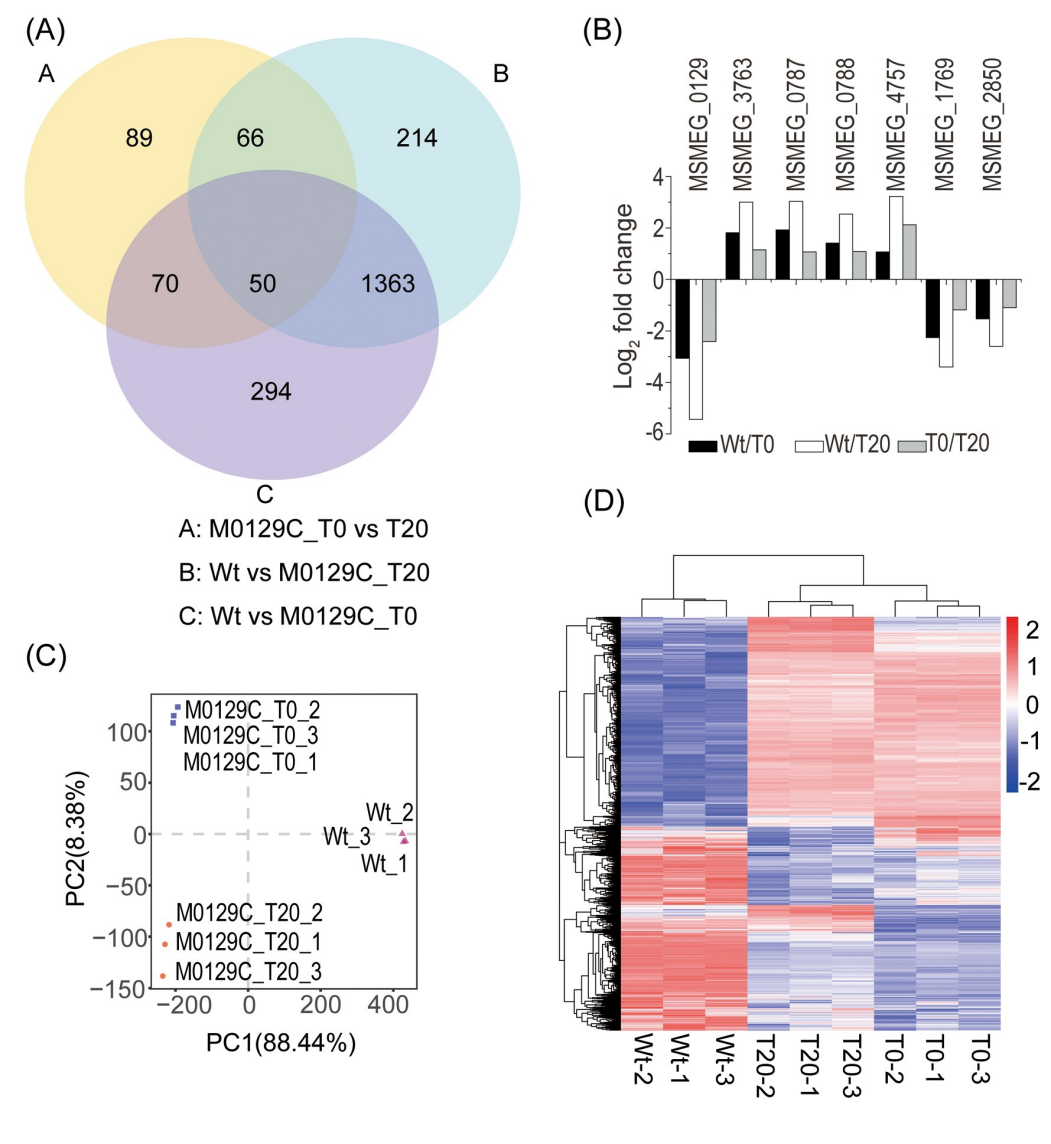
Differentially expressed genes in pair-wise comparisons. (A) Venn diagram showing the numbers of significantly differentially expressed genes (fold change > 2, adjusted p-value < 0.05). (B) Differentially-expressed genes that show similar fold change trends. (C) Principal component analysis of the global expression patterns of the 3 samples. (D) Hierarchical clustering of the 2146 differentially expressed genes (adjusted p-value < 0.05).

To investigate if Mtsp17 functions in transcription regulation, we looked for DEGs in the transcriptomes of the conditional Mtsp17 knockout strain (M0129C) and the wild-type strain (Wt) (S2 Table). Although *mtsp17* was down regulated in the three comparisons (M0129C_T0 versus T20; Wt versus M0129C_T20; Wt versus M0129C_T0), the number of DEGs varied dramatically in each comparison. Overall, 4% of protein-coding genes were differentially-expressed (fold change > 2 and adjusted p-value < 0.05) in the M0129C_T0 versus T20 comparison compared to 24–25% in other 2 comparisons (Table 1). 50 DEGs (fold change > 2) were common to the three comparisons (Fig 1A, S3 Table), and of these, 24 genes (including *mtsp17*) showed the same trends in fold change. We noted that 7 of the 24 genes had homologues in MTB (Fig 1B, Table 2). Similar trends in the expression of DEGs in the 3 comparisons suggest that Mtsp17 plays an important role in regulating gene expression. Fifteen of the 24 genes were up-regulated and 8 were down-regulated, implying that Mtsp17 can act as both an activator and a repressor.

**Table 1:** Numbers of differentially expressed genes.

		M0129C_T0 vs T20	Wt vs M0129C_T20	Wt vs M0129C_T0
*mtsp17* ratio (log_2_ FC)		-2.4	-5.4	-3
Differentially expressed genes (Padj < 0.05, log_2_ FC)	Down > 1	3% (207)	11% (797)	12% (861)
	Up > 1	1% (68)	13% (896)	13% (916)
	< 1	20% (1370)	17% (1150)	18% (1273)
Others		76%	59%	57%

RNA-seq data from 3 replicates of each sample. FC, fold change. Padj, adjusted p-value.

**Table 2:** DEGs common to all three of the pair-wise comparisons.

MSM	MTB	Essential [4]	Description	Functional category
MSMEG_0129	Rv0164	Yes	Mtsp17	Unknown
MSMEG_3763	Rv1686c	No	ABC transporter	Cell wall and cell processes
MSMEG_0787	Rv0411c	No	Extracellular solute-binding protein	Cell wall and cell processes
MSMEG_0788	Rv0412c	No	Hypothetical protein	Cell wall and cell processes
MSMEG_2850	Rv3178	No	Cell entry related family protein	Unknown
MSMEG_1769	Rv3288c	No	UsfY protein	Unknown
MSMEG_4757	Rv2524c	Yes	Fatty acid synthase	Lipid metabolism

### Expression patterns in the two complemented strains were moderately different

Principal component analysis of the global expression patterns of the 3 samples clustered Wt separately from the two complemented strain samples. M0129C_T0 clustered apart from M0129C_T20, with the majority of the variance occurring along PC2 (Fig 1C). Hierarchical clustering of the DEGs (adjusted p-value < 0.05) showed that the gene expression pattern of the M0129C_T20 sample was similar to M0129C_T0 and was different from Wt (Fig 1D). These results indicate that the difference in expression patterns between M0129C_T20 and M0129C_T0 was more moderate than that between the complemented strain and the Wt strain. Comparing the DEGs from the M0129C_T0 and T20 samples is thus more likely to reveal the mechanism by which Mtsp17 regulates gene expression.

### qPCR analysis is consistent with transcriptome data

**Fig 2:**
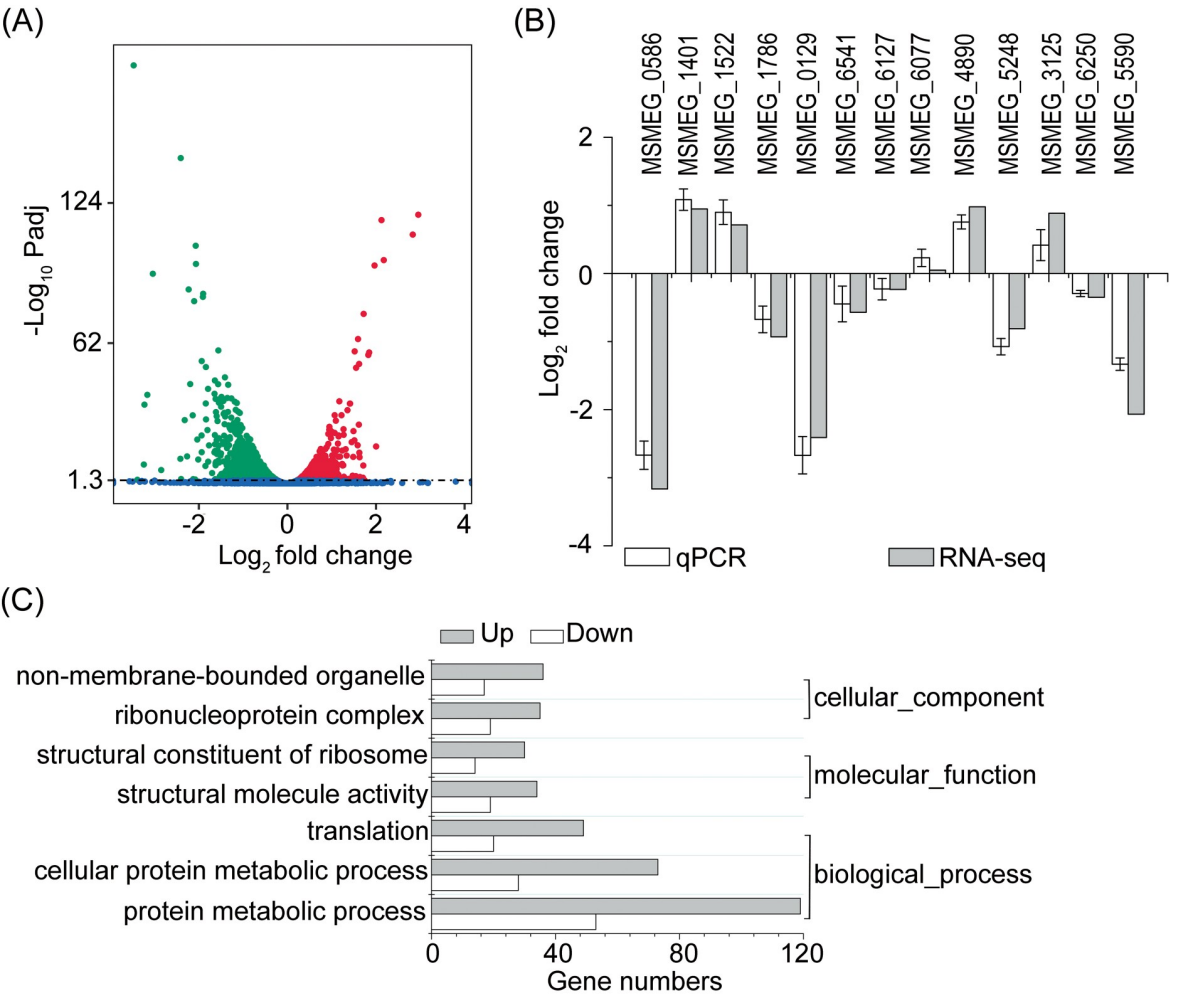
Differentially expressed genes in the M0129C_T0 versus M0129C_T20 comparison. (A) Volcano plot showing differentially expressed genes. Green, down-regulated genes. Red, up-regulated genes. Blue, genes not differentially expressed. Padj, adjusted p-value. (B) qPCR analyses of 13 randomly selected DEGs. (C) Significantly enriched gene ontology (GO) terms (adjusted p-value < 0.05).

Down regulation of Mtsp17 affected the expression of 1645 genes, inducing the expression of 808 genes and repressing that of 837 genes (adjusted p-value < 0.05) (Fig 2A). We randomly selected 13 DEGs for qPCR analyses to verify the gene expression changes observed in our transcriptome data. Trends in fold changes of the qPCR data matched our RNA-seq results (Fig 2B), validating the down-regulated Mtsp17 transcriptome profile generated by RNA sequencing.

### Mtsp17 acts as a transcriptional activator of the *desA1* gene

Gene ontology (GO) analysis of DEGs using GOseq v1.22 indicted that 7 GO terms, including “structural constituent of ribosome” (GO:0003735) and “ribonucleoprotein complex” (GO:0030529), were significantly enriched (adjusted p-value < 0.05) (Fig 2C). Twenty-seven of the 58 ribosomal proteins for which quantitative data was obtained by RNA-seq were differentially-expressed (adjusted p-value < 0.05), however the fold changes of all 27 differentially expressed ribosomal proteins (12 30S ribosomal proteins and 15 50S ribosomal proteins) were less than 2.

**Fig 3:**
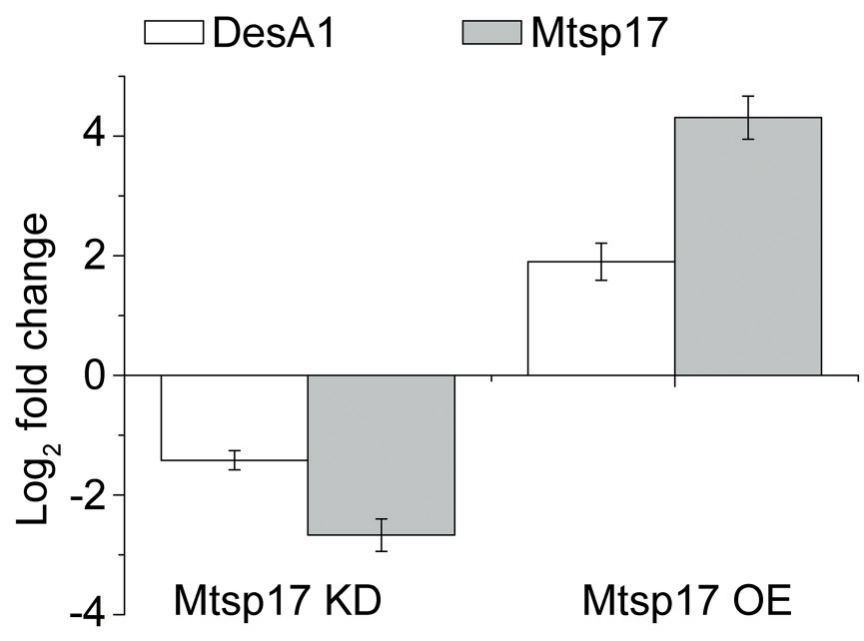
Fold changes of DesA1 and Mtsp17 mRNA. Mtsp17 KD, a conditional Mtsp17 knockout strain, incubated in the absence of the inducer tetracycline for 4 additional hours. Mtsp17 OE, the Mtsp17 over-expression strain.

275 genes were significantly differentially expressed (adjusted p-value < 0.05 and fold change > 2, S4 Table) and 4 of the 275 genes were essential for mycobacterial growth, but only *desA1* (MSMEG_5773) was down-regulated (Log_2_ fold change = -1.62). DesA1 was also positively correlated with Mtsp17 in qPCR data from the conditional Mtsp17 knockout and over-expression strains (Fig 3).

DesA1 is a desaturase involved in mycolic acid biosynthesis in mycobacteria [11]. Changes in mycolic acids are central to the adaptation of MTB to various environments [12,13]. Transcription of *desA1* is known to be regulated by the transcription factor MadR, and is reported to be not affected by other transcription factors [13,14]. MadR-mediated *desA1* repression is evolutionarily conserved and leads to a decrease in mycolic acid biosynthesis and ensuing loss of mycobacterial viability [12]. By analogy, the decline in bacterial growth in Mtsp17 insufficient strains may be due to a decrease in *desA1* transcription.

More transcriptome data related to Mtsp17-*desA1* regulation were searched in the Gene Expression Omnibus (GEO) database and are summarized in Table 3. Overexpression of MadR did not significantly alter levels of *mtsp17* mRNA in either MTB or MSM [13,14]. When 206 transcription factors were overexpressed, *mtsp17* mRNA also showed little change [14]. These phenomena suggest that Mtsp17 may be an upstream factor of transcription factors, and support our hypothesis that Mtsp17 may regulate transcription through a DNA contact-independent mechanism. Under hypoxia, transcriptional change patterns of *madR* and *desA1* were not in accordance with MadR repressing *desA1* and could be explained by Mtsp17 activating *desA1* (Table 3). The above results indicate that Mtsp17 is likely a transcriptional activator of the *desA1* gene.

**Table 3:** Log_2_ fold change values of genes in different pair-wise comparisons.

Genes	MadR overexpression	MadR overexpression	Mtsp17 downregulation	Hypoxia
*madR*	1.48	1.82	0.13	-0.16
*desA1*	-1.46	-1.75	-1.62	-3.25
*mtsp17*	-0.18	0.06	-2.41	-1.53
Species	MSM	MTB	MSM	MTB
GEO*	GSE116027	GSE59086	This study	GSE116353

*the Gene Expression Omnibus (GEO) series accession number.

### Mtsp17 is involved in regulation of the SigF regulon via an anti-SigF antagonist

Transcription in MSM is carried out by a multi-subunit RNA polymerase and one of 28 sigma subunits [15]. The sigma subunit ensures the transcription machinery to initiate transcription of particular genes [16,17]. Sigma factors are post-translationally regulated by a partner switching system (PSS) in which anti-sigma factors sequester the sigma factor from the transcription machinery and anti-sigma factor antagonists lift the repression of sigma factors [18–20].

**Fig 4:**
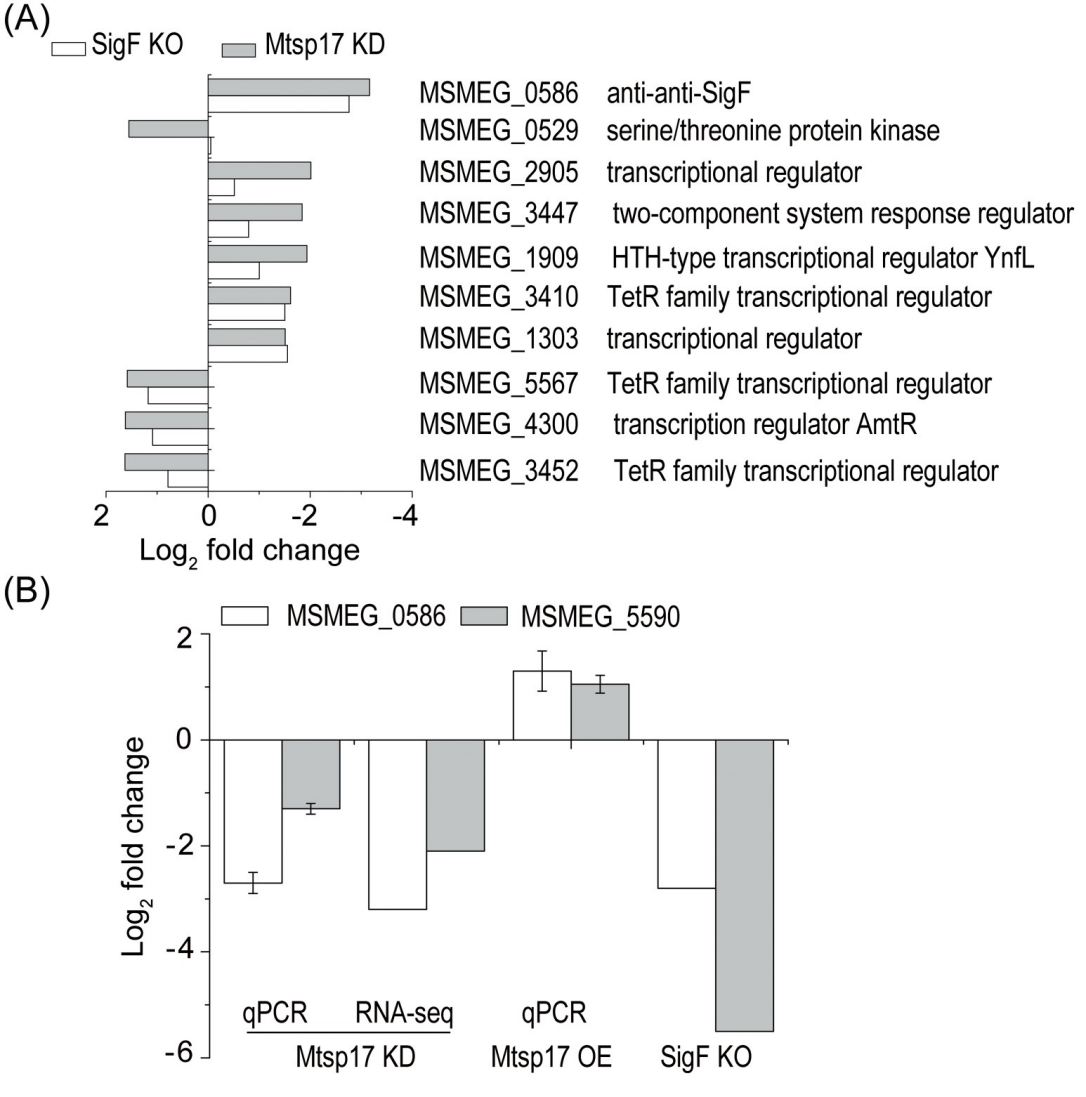
Comparisons of gene expression in Mtsp17 and SigF mutants. (A) 10 differentially expressed genes with log_2_ fold change > 1.5 that are involved in transcriptional regulatory mechanisms and showed the same varying tendency in the SigF knockout (SigF KO) and conditional Mtsp17 knockout (Mtsp17 KD) strains. (B) Fold changes of MSMEG_0586 (anti-anti-SigF) and MSMEG_5590 mRNA in the conditional Mtsp17 knockout (Mtsp17 KD), Mtsp17 over-expression (Mtsp17 OE) and SigF knockout (SigF KO) strains. Data of the SigF knockout strain are from GSE19774.

MSMEG_0586, an anti-SigF antagonist, was one of the top 20 differentially expressed genes, showing an 8.9-fold decrease (log_2_ fold change = -3.16, adjusted p-value = 7.3E-40) in the M0129C_T0 versus M0129C_T20 comparison, while other sigma factors or anti-sigma factors or anti-anti-sigma factors showed log_2_ fold changes of no more than 1.5. MSMEG_0586 is involved in the PSS of the SigF regulatory pathway [20]. In light of significant down-regulation of the anti-SigF antagonist MSMEG_0586 and no obvious change in other SigF PSS factors in the conditional Mtsp17 knockout strain, we hypothesized that transcriptomic changes partially overlapped between the conditional Mtsp17 knockout and the SigF knockout strains. When DEGs in the M0129C_T0 versus M0129C_T20 comparison were compared with DEGs in the SigF knockout versus wildtype comparison [21], 70 of 79 DEGs with log_2_ fold changes > 1.5 and adjusted p-values of < 0.05 showed the same fold change trends (S5 Table). Furthermore, 10 of the 79 DEGs were involved in transcriptional regulatory mechanisms and they all had the same varying tendency in the SigF knockout versus wildtype comparison and M0129C_T0 versus M0129C_T20 comparison, except for MSMEG_0529 (Fig 4A). The above results show that the majority of significantly differentially expressed genes regulated by Mtsp17 belong to the SigF regulon.

To further understand the relationship between Mtsp17 and the SigF regulon, we measured levels of MSMEG_0586 and MSMEG_5590 mRNA in the Mtsp17 over-expression strain by qPCR. MSMEG_5590 was chosen as it belongs to the SigF regulon and was significantly correlated with MSMEG_0586 in the SigF knockout strain (Fig 4B SigF KO). Both MSMEG_0586 and MSMEG_5590 were down-regulated in the conditional Mtsp17 knockout mutant and up-regulated in the Mtsp17 over-expression strain (Fig 4B). The above results indicate that Mtsp17 regulates transcription of MSMEG_0586 and alterations in MSMEG_0586 mRNA levels lead to transcriptional changes in the SigF regulon.

## Discussion

MTB genes that are essential are good targets for drug discovery [22]. Elucidating the mechanism of essential genes is of benefit to the development of more effective vaccines and better drugs. Mtsp17 is an essential protein in both MTB and MSM, but understanding of its function is limited. In the transcriptomic analysis presented here, we have identified genes regulated by Mtsp17 and demonstrated for the first time that a START family protein with a mono domain can regulate transcription in MSM.

The anti-SigF antagonist MSMEG_0586 was one of the top 20 differentially expressed genes in the conditional Mtsp17 knockout mutant and showed an 8.9-fold decrease. Since MSMEG_0586 is as an activator of SigF by post-translational regulation and 70 of 79 DEGs with log_2_ fold changes > 1.5 belong to the SigF regulon (S5 Table), transcriptional changes in these SigF regulon genes can be explained by alterations in MSMEG_0586 expression by Mtsp17 and lead to post-translational regulation of the SigF regulon by the PSS of the SigF regulatory pathway. As both SigF and Mtsp17 are conserved in the Mycobacteria and are transcriptionally regulated in response to nutrient depletion and oxidative stress [10,20], we propose that the regulation of the SigF regulon by Mtsp17 may operate in conjunction with the SigF regulatory pathway during stress responses.

Expression of PknK (MSMEG_0529) showed different trends in terms of fold changes in the Mtsp17 and SigF mutants (Fig 4A). As *pknK* mRNA increases 1.9-fold on SigF over-expression [14] and SigF knockout has no effect on *pknK* mRNA levels [21], we propose the presence of SigF dependent (when SigF is abundant) and SigF independent (when SigF is inadequate) PknK regulatory pathways. Here, down-regulation of *mtsp17* led to a decrease in the anti-SigF antagonist MSMEG_0586 (8.9-fold) and upregulation of *pknK* (2.9-fold). These results correspond to the presence of SigF independent regulation of PknK when SigF is functionally inadequate due to down regulation of the anti-SigF antagonist.

The START protein PCTP interacts with Pax3, a mammalian transcription factor, to regulate gene transcription [23]. In the case of MSM, START protein Mtsp17 interacts with the global transcription factor CarD [10], and the percentage of down-regulated proteins detected here for the Mtsp17 mutant was similar to that reported for the CarD^R47E^ mutant (3% versus 3.1% [24]). However, it is reported that there are no obvious transcriptional changes in DesA1 in CarD mutants, and the anti-SigF antagonist increases in both the loss-of-function (with weakened affinity for RNAP or DNA) and gain-of-function (with increased affinity to RNAP) mutants of CarD [24]. The discrepancy in the transcriptional changes of DesA1 and the anti-SigF antagonist between the Mtsp17 and CarD mutants suggests it is unclear whether CarD plays a key role in this Mtsp17-induced regulatory pathway. Furthermore, we cannot rule out the possibility that other DNA binding proteins are involved in this pathway. Further investigation of Mtsp17 interacting proteins will be necessary to provide a complete understanding of the transcriptional regulatory network of Mtsp17.

This work shows the landscape of Mtsp17 transcriptional regulation and has uncovered important roles of Mtsp17 in the regulatory network of the DesA1 and SigF regulon. As Mtsp17, DesA1 and the SigF regulon are all associated with stress [10,13,25], transcriptional regulation by Mtsp17 likely acts together with other regulatory mechanisms to help coordinate signal transfer under stresses such as nutritional deficiency. Elucidating the detailed mechanism of the Mtsp17 transcriptional regulatory network identified here will require further study.

## Supporting information

S1 Table Primers used in this study. (XLSX)

S2 Table Gene expression profiles of *M*. *smegmatis* mc^2^155 strain (Wt) and *mtsp17*-complemented strains. (XLSX)

S3 Table 50 DEGs (fold change > 2, adjusted p-value < 0.05) overlapping between the three comparisons. (XLSX)

S4 Table 275 significantly differentially expressed genes (fold change > 2, adjusted p-value < 0.05) in the M0129C_T0 versus T20 comparison. (XLSX)

S5 Table 70 DEGs (log_2_ fold change > 1.5, adjusted p-value < 0.05) showing the same change tendency in the M0129C_T0 versus M0129C_T20 comparison and SigF knockout versus wildtype comparison. (XLSX)

## Abbreviations

START: steroidogenic acute regulatory protein-related lipid transfer
qPCR: Real-time fluorescence quantification PCR
MTB: *Mycobacterium tuberculosis*
MSM: *Mycobacterium smegmatis*
CYC/ARO: cyclases/aromatases
PKS: polyketide synthase
RNA-seq: RNA-sequencing
RNAP: RNA polymerase
GO: gene ontology
DEGs: differentially expressed genes
PSS: partner switching system
